# Robust structure measures of metabolic networks that predict prokaryotic optimal growth temperature

**DOI:** 10.1186/s12859-019-3112-y

**Published:** 2019-10-15

**Authors:** Adèle Weber Zendrera, Nataliya Sokolovska, Hédi A. Soula

**Affiliations:** Sorbonne University, INSERM, NutriOmics F75013, France., 91, blvd. de l’Hôpital, 75013 Paris, France

**Keywords:** Metabolic networks reconstruction, Directed graphs, Graph topology, Correlation with environment

## Abstract

**Background:**

Metabolic networks reflect the relationships between metabolites (biomolecules) and the enzymes (proteins), and are of particular interest since they describe all chemical reactions of an organism. The metabolic networks are constructed from the genome sequence of an organism, and the graphs can be used to study fluxes through the reactions, or to relate the graph structure to environmental characteristics and phenotypes. About ten years ago, Takemoto et al. (2007) stated that the structure of prokaryotic metabolic networks represented as undirected graphs, is correlated to their living environment. Although metabolic networks are naturally directed graphs, they are still usually analysed as undirected graphs.

**Results:**

We implemented a pipeline to reconstruct metabolic networks from genome data and confirmed some of the results of Takemoto et al. (2007) with today data using up-to-date databases. However, Takemoto et al. (2007) used only a fraction of all available enzymes from the genome and taking into account all the enzymes we fail to reproduce the main results. Therefore, we introduce three robust measures on directed representations of graphs, which lead to similar results regardless of the method of network reconstruction. We show that the size of the largest strongly connected component, the flow hierarchy and the Laplacian spectrum are strongly correlated to the environmental conditions.

**Conclusions:**

We found a significant negative correlation between the size of the largest strongly connected component (a cycle) and the optimal growth temperature of the considered prokaryotes. This relationship holds true for the spectrum, high temperature being associated with lower eigenvalues. The hierarchy flow shows a negative correlation with optimal growth temperature. This suggests that the dynamical properties of the network are dependant on environmental factors.

## Background

All living organisms rely on chemical reactions to exist, and the set of these life-sustaining chemical transformations is defined as metabolism. Because these reactions are mostly catalysed—accelerated—by enzymes, the transformation of organic molecules (substrates) into other chemicals (products) can directly be mapped by the enzyme set.

The development of metabolic databases such as KEGG [1] linking enzymes to their reaction pair —substrates/products— allows us to explore the structure of metabolism in general, and to investigate the structures of the metabolic graphs of particular organisms [2].

Flow is an inherent concept of metabolic reactions, going from substrates to products which then become substrates for other reactions. Directed graphs are therefore a natural way to model enzymes and chemical reactions [3]. The metabolic network of an organism is defined as the whole set of metabolic pathways. Since such a network is a (directed) graph, the elements of graph theory can be applied to study its properties. Understanding network topologies and their physical, chemical, and biological constraints is critical to decipher the function and evolution of cellular networks [4].

**Fig. 1:**
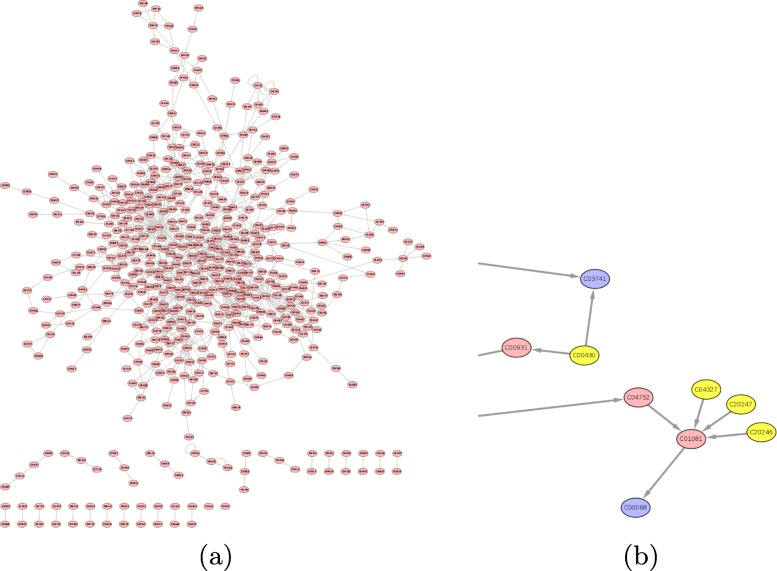
Metabolic networks complexity. **a** Metabolic network of archaea *Methanopyrus kandleri*; **b** Zoom in the network of archaea *Methanopyrus kandleri*: metabolites without predecessors are shown in yellow, and metabolites without successors are shown in blue. The code C followed by 5 digits are compound codes in the KEGG database

We focus on a metabolite-centered representation where the nodes of a graph are metabolites, and they are connected if an enzymatic reaction converting one metabolite into another exists. It is then simple to create metabolic networks that describe all chemical reactions of one or multiple organism(s) as a graph. Figure 1 provides an example of the topology of a directed graph for a bacterium. In a directed graph, some nodes are end-points (shown in blue), and some nodes are starting-points (in yellow). To keep our flow analogy in play, starting points compounds will be an input (e.g. from the medium) whereas end points are ’final’ products of the complete pathways.

Recent studies take two main directions in the analysis of metabolic networks. The first one heavily relies on graph structural measures such as degree distribution, clustering coefficient, path length, and centrality [5–7]. The second venue of research is based on the analysis of biomass dynamics inherent to metabolic networks, by trying to predict steady-state flux distributions. Various constraint-based techniques exist to solve this problem, e.g., flux balance techniques (FBA) [8].

It was reported by several studies (e.g., [9–12]) that the compounds of metabolic networks, the flow of substrates and products, and the overall pathway organisation are correlated to environmental variables and to phenotypical traits. The aim of these studies was to discover similarities and differences in the structural and functional properties of various organisms. It was noticed [9] that evolutionary changes in metabolic networks are mostly due to adaptation to changing conditions. So, Takemoto et al. [11] made an attempt to explore correlations between several structural properties of metabolic networks (such as edge density, power law degree exponent, clustering coefficient, and subgraph concentration) for 113 prokaryotes to their optimal growth temperature.

Ideally, metabolic networks require complex representations such as hypergraphs, since reactions in metabolic networks convert multiple reaction inputs into multiple outputs using other components [13, 14]. However, a reduced representation and algorithms on graphs can facilitate the analysis by addressing the fundamental biological concepts.

A number of graph theory approaches were proposed to study relations between the structure of metabolic networks and the environment. So, Borenstein et al. [10] stated that species whose environment are highly-predictable tend to have smaller sets of compounds that are exogenously acquired than those who live in variable conditions.

The network topology determines network functions [15], and the topology of a metabolic network is important in predicting the viability of mutant strains.

Metabolic networks are known to be extremely heterogeneous, and two networks of two different organisms are quite different [16]. At the same time, the metabolic networks were shown to be robust in the sense that elimination of several central nodes does not modify the functions of the networks [16]. A graph-based method to identify all minimal reaction sets in a metabolic network was considered in [17].

Our main motivation is to explore the structure of directed metabolic graphs of bacteria, and to relate it to phenotypes. In our experiments, we consider prokaryotic optimal growth temperature as a phenotype. In this article, our contribution is:

- We reconstruct metabolic networks for species considered by [11] in addition to several species that will increase the number of species in the growth temperature classes which were represented by too few species.
- We build and explore undirected and directed metabolic graphs; including all KEGG enzymes for the species or only those found in the so-called KEGG pathways.
- We propose to apply robust measures on directed complex graphs, namely largest strongly connected component, flux hierarchy and Laplacian spectrum, and we relate these measures to the environmental conditions.
- In our experiments, we have confirmed the results of [11], and we discuss the newly introduced metrics.

## Results

### Confirmation of the state-of-the art results of Takemoto et al. (2007)

In the study conducted by [11], undirected substrate graphs were constructed using KEGG metabolic pathways for 113 prokaryotes from four different growth temperature classes (hyperthermophiles, thermophiles, mesophiles, and psychrophiles). They considered several properties of undirected graphs, and analysed the correlation between the graph properties and the optimal growth temperatures of the organisms. We implemented and tested three measures from their study: edge density, maximum likelihood estimate of degree exponent, and average clustering coefficient.

Since more than ten years have gone by, the databases have evolved significantly. We focused on directed graphs, and we reconstructed the metabolic networks without using pathways directly (see Methods). We obtained graphs for 100 out of the 113 species, filtering out nodes in such a way that we had metabolic reactions from known pathways only. We also added 128 additional species.

There are two main differences between our experiments and the ones from [11]. We use more species in our experiments, and we consider directed graphs. We still confirm most of the results from [11].

**Fig. 2:**
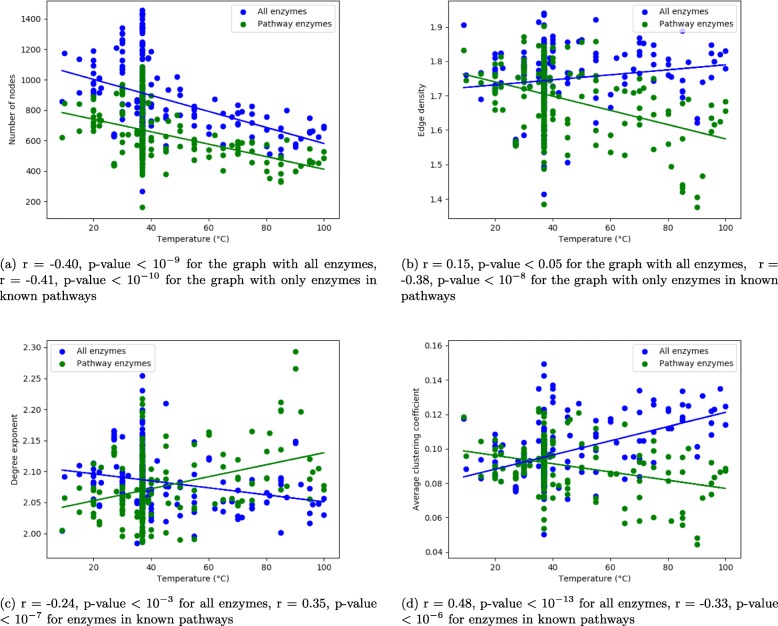
Impact of additional enzymes: **a** the Pearson’s correlation between the number of nodes and the optimal growth temperature; **b** the correlation between the edge density and the optimal growth temperature; **c** the correlation between the degree exponent and the optimal growth temperature; and **d** the correlation of the average clustering coefficient and the optimal growth temperature. All these measures are applied on undirected substrate graphs without ubiquitous metabolites

We apply a linear regression to estimate relation between the number of nodes in a metabolic network and the optimal growth temperature and deduce Pearson’s correlation. We consider a correlation between the number of nodes and the optimal growth temperature, a correlation between edge density and optimal growth temperature, a correlation between degree exponent and optimal growth temperature, and a correlation of average clustering coefficient and optimal growth temperature. All these measures are applied to undirected substrate graphs without ubiquitous metabolites, to be as close to [11] as possible. As we can see from Fig. 2 (green lines), there is a significant negative correlation between the number of nodes and the temperature shown on Fig. 2a, and a negative correlation between edge density and the temperature illustrated on Fig. 2b. As shown on Fig. 2c and d, we find a significant positive correlation for the degree exponent estimate, and significant negative correlation for the average clustering coefficient with the optimal growth temperature of the species.

In general, we discovered the same tendencies as [11].

### Undirected graphs reconstructed from all enzymes and the impacts on graph properties

Here we compare the influence of taking all KEGG enzymes for a species with enzymes in known KEGG pathways only. Our method to build the metabolic graphs is different from [11] in that we consider all reactions that can be deduced from the species genes, and not only enzymes involved in known pathways. We have, therefore, found additional enzymes for all species.

On Fig. 2a, we notice a strong bias related to the number of nodes in respect to the growth temperature. Hence, ideally, graph properties are to be normalised by the number of nodes.

We wish to analyse the impact of the additional reactions. We observe a complete loss of the tendencies, what is shown in blue on Fig. 2b, c and d. All the correlations are inverted, albeit with lower correlations except for the average clustering coefficient, which has a stronger correlation than what [11] found.

We found a significant positive correlation between the proportion of enzymes that are not in a pathway and optimal growth temperature (Pearson’s r = 0.26, data not shown), meaning that more new enzymes—edges—are added for thermophiles than non-thermophiles, adding more edges and therefore likely causing the correlation inversions.

When removing up to 40% of random nodes in the graphs with all enzymes, the trends stay significantly correlated. This means that the pathway enzymes are specific and greatly modify the graph structure.

These differences could be due to a bias in KEGG pathways for hyperthermophiles, that could have less annotated and curated pathways than its more well-studied counterparts, and thus more enzymes not associated to pathways. Another hypothesis is that this difference could be explained by noise, since the number of nodes for hyperthermophiles is the smallest.

### Robust directed measures to analyse cycles of metabolic networks

We focus on directed metabolic networks, and we are interested in finding relevant measures on directed graphs that can explain correlation with environmental variables such as optimal growth temperature. We propose robust measures to analyse substrate graphs.

We considered two measures to study cycles in networks, the size of the largest strongly connected component which corresponds to the biggest cycle in a graph, and the flow hierarchy (see Methods). The flow hierarchy is defined as the number of nodes in a component that is not a part of the largest strongly connected component. So, the two explored measures are closely related.

**Fig. 3:**
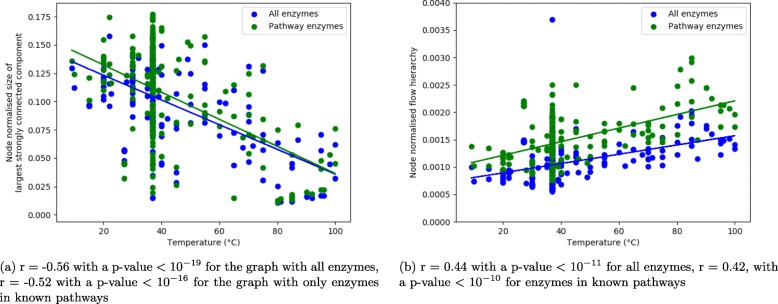
Robust measures on directed graphs: **a** Pearson’s correlation between the node normalised size of the largest strongly connected component and the optimal growth temperature; and **b** Pearson’s correlation between the node normalised hierarchy flow and the optimal growth temperature. These measures were applied to directed substrate graphs for all 228 species, without ubiquitous metabolites

As shown in Fig. 3, we found a significant negative Pearson’s correlation between the size of the largest strongly connected component, normalised by the number of nodes, and the optimal growth temperature. We have also observed a significant positive Pearson’s correlation between the node normalised flow hierarchy and the optimal growth temperature. These tendencies have been found for substrate graphs built with all enzymes and for substrate graphs with enzymes in known pathways: these measures are consistent in both cases, with similar correlations thus becoming robust measures to analyse correlation between metabolic network structure and environmental conditions. We consider that these measures are potentially more relevant to describe metabolic networks as they reflect directed graphs properties.

### Metabolic network Laplacian eigenvalues

We tested another directed network structural property to study connectivity. A temperature class network yields different graph structural properties related to connectivity, but the underlying description of these graphs is a compound flow. These properties are associated to the ’speed’ of reactions and can be assessed using the spectrum of the network [18].

More precisely, for each species’ graph we computed the adjacency matrix containing all recorded compounds among all species yielding a matrix *A* of dimensions 3194×3194. We compute the Laplacian matrix $\mathcal {L}$ and extract its spectra (the ordered from high to low list of eigenvalues of $\mathcal {L}$). These values must be comprised within the interval [0,2]. For example, for a star graph with *n* vertices, the eigenvalues are 0, 1 (with multiplicity *n*−2) and 2, and for the cycle on *n* vertices the eigenvalues are $1-\cos \left (\frac {2\pi k}{n}\right)$ for 0≤*k*<*n*.

**Fig. 4:**
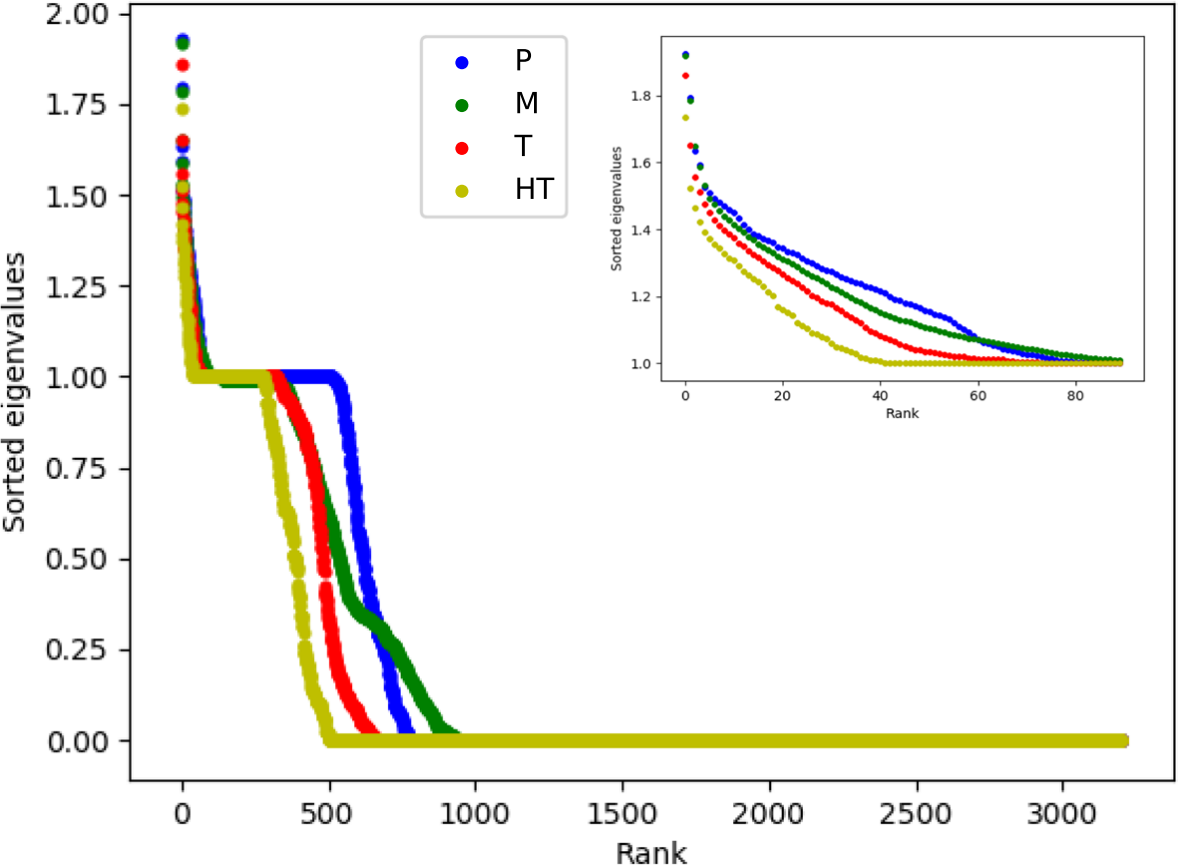
Averaged sorted eigenvalues of the Laplacian matrix of reaction graph for all groups. In insert a close-up for the first 90 eigenvalues

We computed this spectrum for each species, and estimated the average within the same temperature group (see values in Additional file 2). The results are shown on Fig. 4. The results illustrate clearly that for a temperature class structuration, a higher temperature is associated with lower eigenvalues.

In Fig. 4 we can see steps that can be observed at value 1 and 0. Eigenvalues of 0 have a multiplicity equal to the number of connected components in the graph, but also since we computed the adjacency matrices as the union of all compounds for all species, eigenvalues of 0 also reflect nodes that do not actually exist in a given graph but exist in another (Fig. 2a shows that the largest networks have a maximum of around 1500 nodes whilst Fig. 4 shows 3200 eigenvalues). These non-existent nodes are therefore considered as isolated nodes and counted as a connected component, having an eigenvalue of 0.

On the other hand, eigenvalues of value 1 with eigenvectors summing to 0 correspond to a particular network pattern :

- Source nodes with at least one target with a single in-degree (whose only predecessor is the source node),
- The said targets.

Source nodes are nodes without predecessors (in-degree of 0).

## Discussion

We have observed that prokaryotic metabolic network properties can correlate with environmental phenotypes, namely with the optimal growth temperature in our study.

First of all, we confirmed the results of [11] that dates more than ten years back: a negative correlation between edge density and optimal growth temperature and between average clustering coefficient and optimal growth temperature, and a positive correlation between maximum likelihood estimate of degree exponent and optimal growth temperature. This clearly shows the robustness of the data from the KEGG database, even though some data has been modified, and some new data has been added. On another hand, the results of our experiments also illustrate the validity of the results of [11]. Although the amount of species in our experiments is doubled compared to the number of prokaryotic species in [11], the trends are still the same.

These results hold for a particular subset of known metabolic data of the species. We consider important to take all available data (enzymes) into account. However, in the case where all enzymes associated to the species in KEGG are taken into account, these results do not hold anymore: they change sign of the correlation. The origins of these inversions are still unclear, but we believe that it could be due to biases in KEGG for non-mesophilic species (especially for thermophilic species).

We believe a directed network representation is more appropriate to model metabolism, so we looked for directed topological properties that were robust for the different reconstruction protocols. We tested directed graph structural properties related to cycles (largest strongly connected component and flow hierarchy) and to connectivity and flow (Laplacian spectrum).

We found that when there are less nodes involved in the largest strongly connected component (cycle), it is linked to higher optimal growth temperatures, and there are more nodes outside of the cycle that are still part of the weakly connected component, which is the measure of hierarchy flow. When more nodes are involved in the largest strongly connected component, it is linked to lower optimal growth temperatures, and there are less nodes outside the cycle that are still part of the weakly connected component. This is valid no matter if all enzymes are considered or only pathway enzymes.

We explored the most common nodes of the largest strongly connected components, and we found several metabolites such as L-glutamate and L-glutamine (found in 213 out of 228 species), pyruvate (found in 213 from 228 species), phosphoenolpyruvate (209/228), carbamate (208/228) and some others which are molecules involved in the most basic cell metabolism, and that may imply the primordial and basic functions of these metabolic cycles.

Amino acid substitutions are reported to be more deleterious for thermophiles than non-thermophiles [19], implying less variability in enzymes, thus less enzymes, explaining the negative correlation between the number of nodes in our graphs and optimal growth temperature (Fig. 2a). We can hypothesize that for this very reason, the set of core enzymes and metabolites of the metabolism, which could be represented by the largest strongly connected component, also represents a smaller fraction of nodes because of the greater evolutionary pressure given by temperature. Consequently, as the fraction of nodes in the largest strongly connected component is smaller for thermophiles, the fraction of nodes for the flow hierarchy is larger.

On another hand, we see that Laplacian eigenvalues are higher for prokaryotes that preferentially grow in colder environments, showing more particular patterns of connectivity and flow in their networks.

Other directed graph topological properties were tested, with some having significant correlations with optimal growth temperature for both reconstructions, such as the fraction of nodes with an in-degree of 0 (starting-point nodes, input metabolites) or the fraction with an out-degree of 0 (end-point nodes, output metabolites), having both positive correlations with temperature (data not shown), or also the number of some of the triads among the 16 possible triads in a directed network also show significant correlations, positive and negative (data not shown). All of this shows the clear link between the immediate environment and the metabolism of a given species, and can be looked into in different contexts and environments.

To integrate directed graphs and bring it a step further, an interesting future research avenue would be to study the differences, or complementarities between community graphs and single organism graphs, as well as differences in their largest strongly connected components and other directed structural properties.

An important point has to however be made on the direction of chemical reactions. In this work, we fixed the directions of reactions as found in the KEGG database. However, there might be some chemical reactions happening in the opposite direction than the one fixed in KEGG. We believe that it may be interesting to infer directionality of reactions, since homeostasis is extremely important for organisms, and it is regulated, e.g., by enzymes. There is a need to study this problem, for example through thermodynamics, and it would be promising to study the flux of our graphs, in particular from observational data to investigate the dynamics of the biomass.

## Conclusions

We have reproduced the results of [11], and we state that the results mostly hold even with the evolution of the KEGG database, and even while significantly increasing the number of species in the data set. We have found a positive correlation between the degree exponent estimate and optimal growth temperature, and a negative correlation between the edge density and the temperature and between average clustering coefficient and the temperature.

We have noticed that when we include all KEGG enzymes we could find for a species into metabolic networks, and not only the enzymes from KEGG pathways, the results do not hold anymore.

We propose three directed graph measures, namely, the size of the largest strongly connected component, the flow hierarchy, and the Laplacian spectrum. We have shown that these measures are robust for all considered graphs, and they correlate respectively negatively, positively and negatively to the optimal growth temperature. In all our experiments, we have observed strong links between environmental phenotypes and graph structure.

We have also developed a pipeline to reconstruct metabolic networks taking into account all enzymes. We compared the results of our pipeline to the state-of-the-art results of [11], and we can state that our pipeline yields very reasonable results.

We are currently investigating how robust the metabolic networks are against structural modifications. Finding causal directions from purely observational data is another open challenge.

## Methods

### Prokaryotic species

Our data set contains 228 prokaryotic species where 100 species are from the databased used by [11]. We decided to increase the number of species in our experiments, since the number of bacteria in three growth temperature classes was too small (1 psychrophile, 9 hyperthermophiles, and 9 thermophiles). We added 52 species from the Bacterial Diversity metadatabase (BacDive) [20], chosen according to their growth temperature class. We also added 76 mesophilic species from the Human Pan-Microbe Communities (HPMC) database [21]. We obtained the following distribution over four growth temperature classes (from hot to cold): 19 hyperthermophiles (HT), 35 thermophiles (T), 158 mesophiles (M), and 16 psychrophiles (P). Mesophiles are the most well-studied species, thus biasing databases towards these species and explaining the temperature distribution of our species. Hyperthermophilic species are species whose optimal temperature is above 80^*o*^C, thermophilic species are ones with the optimal temperature between 50^*o*^C and 70^*o*^C, mesophilic species live in the range between 20^*o*^C and 45^*o*^C, and psychrophilic species prefer an environment between −20^*o*^C and 10^*o*^C.

### Metabolic network reconstruction

**Fig. 5:**
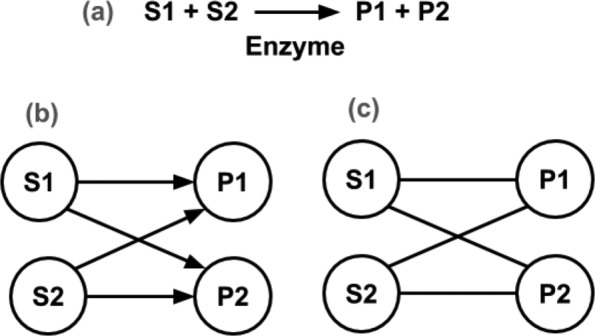
Metabolic network representation: **a** enzymatic reaction, S1 and S2 the substrates, and P1 and P2 the products; **b** a directed graph representation; **c** an undirected graph representation

There exist a number of ways to produce metabolic networks from chemical reactions. The nodes of such a metabolic reconstruction can be metabolites (small molecules, substrates, and products of the enzymes), or enzymes. We have built directed and undirected substrate graphs which are metabolite-centered graphs where each substrate is a node and is linked to each product of a metabolic reaction for a given species. Therefore, edges are enzymatic reactions linking substrates to products. Figure 5 sketches a metabolic reaction, a directed graph, and an undirected metabolic network.

**Fig. 6:**
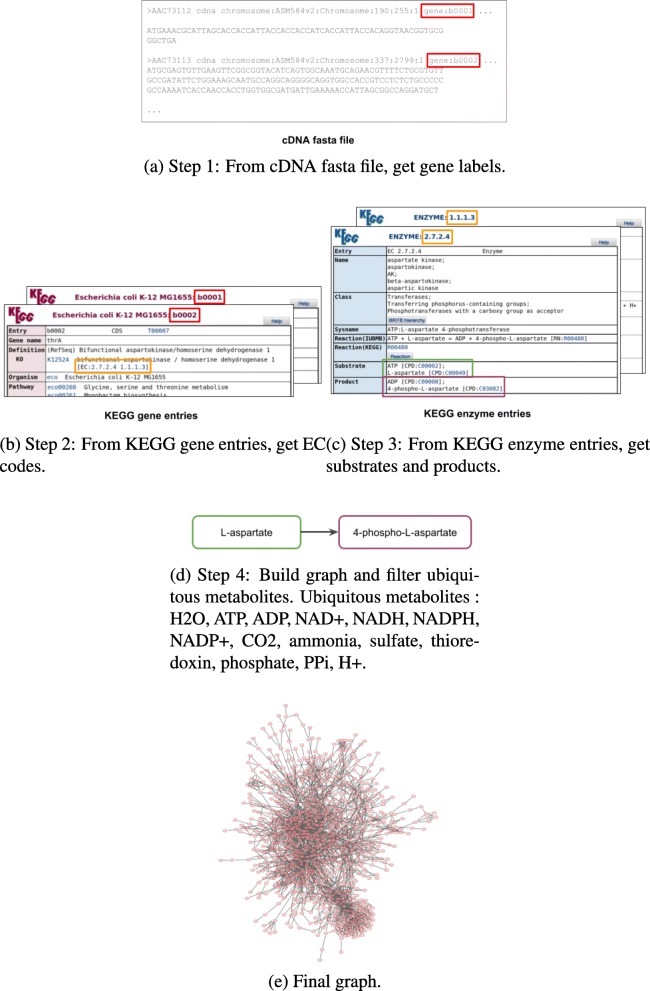
Our network reconstruction procedure. **a** Step 1: From cDNA fasta file, get gene labels, **b** Step 2: From KEGG gene entries, get EC codes, **c** Step 3: From KEGG enzyme entries, get substrates and products, **d** Step 4: Build graph and filter ubiquitous metabolites. Ubiquitous metabolites : H2O, ATP, ADP, NAD+, NADH, NADPH, NADP+, CO2, ammonia, sulfate, thioredoxin, phosphate, PPi, H+, **e** Final graph

To build the metabolic graphs, we downloaded Ensembl or GenBank cDNA FASTA files for the 228 species. Figure 6 shows the reconstruction procedure. We retrieved gene labels from the FASTA files, see Fig. 6a. We then consulted the Kyoto Encyclopedia of Genes and Genomes (KEGG, [22]) database. With the KEGG code for a species and the gene labels, we found the species gene entries, and we extracted all enzyme commission codes (ECs) if the codes were found in complete form, i.e., no hyphen was present in the code. This step is shown on Fig. 6b. We then extracted all substrates and products from the KEGG enzyme entries (Fig. 6c), and we built directed and undirected substrate graphs, which is illustrated by Fig. 6d and e. We excluded 13 species out of the 113 species from [11] because we could not find gene names in the cDNA FASTA files, or the gene names did not match to the KEGG species code, or the species entry (and code) in KEGG simply did not exist anymore.

A common practice in metabolic network reconstruction is to exclude ubiquitous metabolites to make the network more relevant biologically, and because of the great impact on network structure. There is no strict consensus on ubiquitous metabolites, however, the metabolites used as carriers for transferring electrons and common functional groups are regarded as ubiquitous metabolites [23]. Similarly to [11], we defined 13 ubiquitous metabolites: H_2_O, ATP, ADP, NAD^+^, NADH, NADPH, CO_2_, ammonia, sulfate, thioredoxin, phosphate, pyrophosphate (PP_i_), and H^+^. We also consider NADP^+^ to be a ubiquitous metabolite. All these metabolites do not appear in our graphs.

Note that in [11] they directly downloaded the metabolic pathways of the prokaryotes from the KEGG which are curated networks and are, therefore, different from the networks found with the full enzyme set of the prokaryotes. In order to replicate as accurately as possible their results, we also built graphs without enzymes that do not have an associated KEGG pathway (without PATHWAY field in the KEGG enzyme entry).

Indeed, our main objective is to assess bacterial metabolic systems without any a priori knowledge, and therefore keep as much information as possible, which is why we keep all enzymes that can be deduced from the genome. This means that we have kept most inorganic compounds and generic reactions. We therefore may have less metabolic information regarding some nodes when considering generic reactions, but also more information as more data from the database is considered and as all substrates and products are included (pathways sometimes only show the main reactants and not all of them).

For the directed reconstructed graphs, the default direction of the KEGG reaction was used, which is the direction of the catalytic reaction (substrates and products are specified). It is the direction in which the flow of biomass is expected.

Our networks were reconstructed on April 2019, a description of the species and the networks can be found in Additional file 1.

### Optimal growth temperatures

For the species also considered by [11], we got the optimal growth temperatures from the supplementary material provided with their article. The data originally came from the Prokaryotic Growth Temperature Database (PGTdb) [24]. The access to the PGTdb was not available since we started performing our experiments and later, so, the optimal growth temperature and the growth temperature classes for the rest of the species were taken from the BacDive database [20]. For the species whose optimal growth temperature was given as an interval in the BacDive database, we used the average value of the interval. For the species from the Human Pan-Microbe Communities (HPMC) database, the optimal growth temperature was fixed to 37^∘^*C*.

### Measures on directed and undirected graphs

#### Edge density for undirected graphs

Here we use the definition provided in [11] for the edge density for an undirected graph:

1 $$\begin{array}{*{20}l} \text{Edge density}= \frac{E}{N}, \end{array} $$

where *E* is the total number of edges, and *N* is the total number of nodes.

#### Maximum likelihood estimate of degree exponent

We follow the definition given by [11]. We assume that the degree distribution *P*(*k*) of our graph follows a power law *k*^- *γ*^. The number of connections *k* of a node is called *degree* of a node, and the degree distribution is the degrees of nodes over the whole graph. An estimate via maximum likelihood of the degree exponent *γ* is as follows:

2 $$\begin{array}{*{20}l} \gamma = 1 + N \times \left[\sum \limits_{i=1}^{N} \ln\frac{k_{i}}{k_{min}}\right]^{-1}, \end{array} $$

where *N* is the number of nodes in the network, *k*_*i*_ is the degree of node *i* and *k*_min_ is the smallest degree in the metabolic network. We do not take into account nodes with null degrees for this measure.

#### Average clustering coefficient

Here we have used an approximation of the average clustering coefficient. The local clustering coefficient of a node *i* in an undirected graph *G* is defined as:

3 $$\begin{array}{*{20}l} C_{i} = \frac{M_{i}}{M_{possible}}, \end{array} $$

where *M*_*i*_ is the number of triangles formed by a node and two of its neighbours, and *M*_*possible*_ is the number of all possible triangles that could be formed with this node’s neighbourhood. The average clustering coefficient corresponds to an average value of local clustering coefficients over all nodes. The approximation we have applied is the one proposed by [25] where the action of choosing a node at random and checking whether its two random neighbours are connected is repeated *n* times (we have taken *n*=1000).

The average clustering coefficient $\overline {C}$ then becomes:

4 $$\begin{array}{*{20}l} \overline{C} = \frac{M}{n}, \end{array} $$

where *M* is the number of triangles found, and *n* is the number of trials.

#### Node-normalised size of the largest strongly connected component

**Fig. 7:**
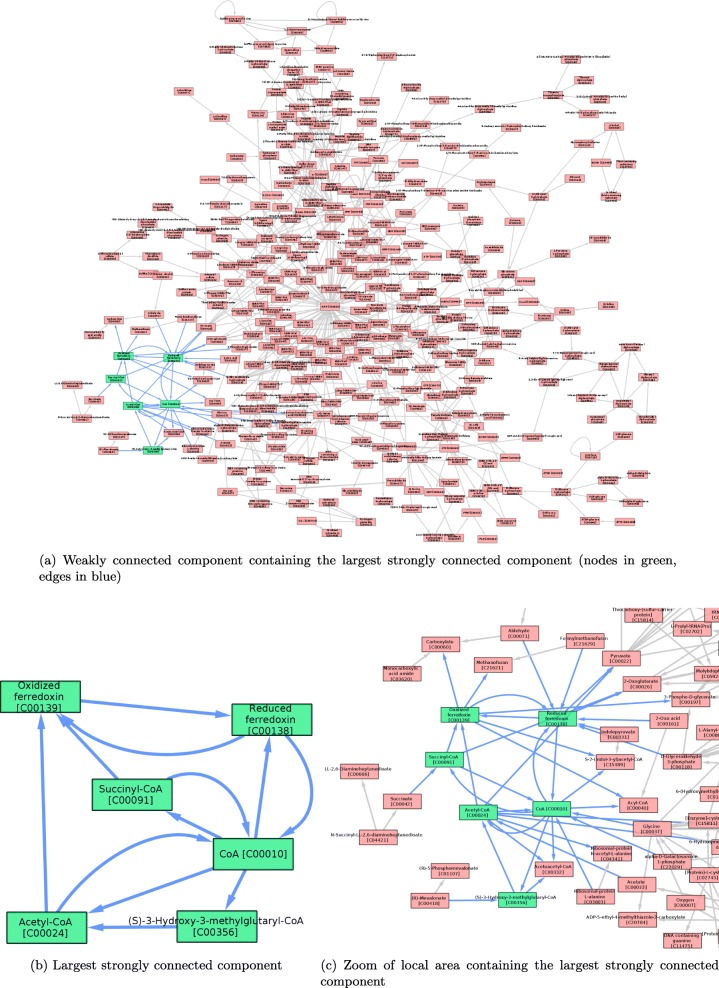
Visualization of the largest strongly connected component and weakly connected component for *Desulfurococcus amylolyticus 1221n*. **a** Weakly connected component containing the largest strongly connected component (nodes in green, edges in blue), **b** Largest strongly connected component, **c** Zoom of local area containing the largest strongly connected component

The largest strongly connected component corresponds to the largest partition of path equivalent nodes in a directed graph. Path equivalence is the property of having a path from node *v* to node *w*, and a path from *w* to *v* in a given graph *G* [26]. Therefore, the node-normalised size of the largest strongly connected component is the number of nodes of the largest strongly connected component divided by the number of nodes. Note that applying this definition, the strongly connected components are cycles. An example of the largest strongly connected component for *Desulfurococcus amylolyticus 1221n* is shown on Fig. 7. The number of nodes in the largest strongly connected component might be small compared to the number of all nodes in a graph (in the example it is 6 out of 340 nodes).

#### Node-normalised hierarchy flow

**Fig. 8:**
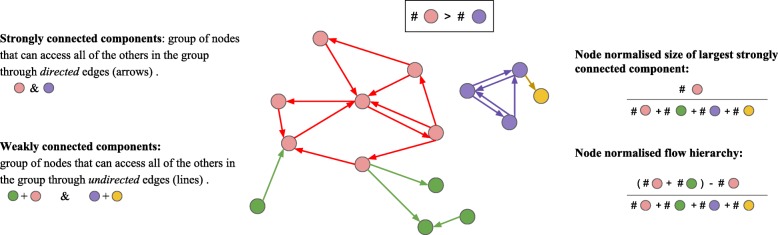
The largest strongly connected component and the weakly connected component of a graph: an intuition behind the measures

A weakly connected component is also a property of directed graphs. It is defined as a group of nodes where each node *v* and *w* are connected via an undirected path. We have defined the concept of flow hierarchy as the number of nodes that do not participate in the largest strongly connected component. Hierarchy flow can therefore be deduced from the subtraction of the strongly connected component from the weakly connected component. To be precise, we take the largest strongly connected component and the weakly connected component containing it, and we then divide the remainder nodes by the number of nodes in the graph (normalization). This procedure is drafted on Fig. 8, and can be observed in the example of *Desulfurococcus amylolyticus 1221n* in Fig. 7.

#### Laplacian matrix and spectrum

We collected the set of all compounds described in the different graphs to create a standardized adjacency matrix *A* for each species indexed by these vertices *A*_*xy*_=1 if a *directed* edge exists from *x* to *y*. By construction, *A* is not usually symmetric. We compute the Laplacian matrix $\mathcal {L}$ also indexed by the vertices whose sum over the columns are equal to zero and *L*_*xy*_=−*A*_*xy*_ if *x*≠*y*. We computed the spectrum —the list of eigenvalues— of $\mathcal {L}$ and ordered it by highest to lowest. We computed the average of this sorted vector for all species within a temperature class.

## Supplementary information

**Additional file 1** This file contains our prokaryotes and structural properties information in an XLSX format. It contains the species name, KEGG species code, optimal growth temperature, temperature class, number of nodes for all enzyme graphs, number of nodes for pathway graphs, the number of edges for all enzyme graph, number of edges for the pathway graph, the average clustering coefficient for the all enzyme graphs, the average clustering coefficient for the pathway graphs, the degree exponent for the all enzyme graphs, the degree exponent for the pathway graphs, the edge density for the all enzyme graphs, the edge density for the pathway graphs, the node normalised flow hierarchy for the all enzyme graphs, the node normalised flow hierarchy for the pathway graphs, the node normalised size of the largest strongly connected component for the all enzyme graphs, the node normalised size of the largest strongly connected component for the pathway graphs.

**Additional file 2** This file contains the average Laplacian spectrum per temperature class, sorted in decreasing order. The Laplacian matrix size is the number of nodes in the union of nodes for all our networks.

## Data Availability

The data used in the numerical experiments are publicly available. KEGG database is available at https://www.kegg.jp/, BacDive database can be accessed at https://bacdive.dsmz.de/, and HPMC database at http://www.hpmcd.org/. PGT database is no longer available, growth temperatures were taken from [11] additional file.

## Abbreviations

ADP: Adenosine Diphosphate
ATP: Adenosine Triphosphate
BacDive: Bacterial Diversity metadatabase
cDNA: Complementary Deoxyribonucleic Acid
CO_2_: Carbon dioxide
CoA: Coenzyme A
EC: Enzyme Commission number
FBA: Flux Balance Analysis
H^+^: Proton
H_2_O: Water
HPMC: Human Pan-Microbe Communities database
HT: Hyperthermophiles
KEGG: Kyoto Encyclopedia of Genes and Genomes database
M: Mesophiles
NAD^+^: Oxidized Nicotinamide Adenine Dinucleotide
NADH: Reduced Nicotinamide Adenine Dinucleotide
NADP^+^: Oxidized Nicotinamide Adenine Dinucleotide Phosphate
NADPH: Reduced Nicotinamide Adenine Dinucleotide Phosphate
P: Psychrophiles
PGTdb: Prokaryotic Growth Temperature database
PP_i_: Pyrophosphate
T: Thermophiles
